# Palliative Role of Zamzam Water against Cyclosporine-Induced Nephrotoxicity through Modulating Autophagy and Apoptosis Crosstalk

**DOI:** 10.3390/toxics11040377

**Published:** 2023-04-16

**Authors:** Medhat Taha, Sara T. Elazab, Tourki A. S. Baokbah, Abdullah G. Al-Kushi, Mohamed Ezzat Mahmoud, Omer Abdelbagi, Naeem F. Qusty, Ibrahim El-Shenbaby, Omar Babateen, Alaa. M. Badawy, Mohie Mahmoud Ibrahim

**Affiliations:** 1 Department of Anatomy and Embryology, Faculty of Medicine, Mansoura University, Mansoura 35516, Egypt; 2 Department of Anatomy, Al-Qunfudah Medical College, Umm Al-Qura University, Al-Qunfudah 28814, Saudi Arabia; 3 Department of Pharmacology, Faculty of Veterinary Medicine, Mansoura University, Mansoura 35516, Egypt; 4 Department of Medical Emergency Services, College of Health Sciences-AlQunfudah, Umm Al-Qura University, Al-Qunfudah 28814, Saudi Arabia; 5 Department of Human Anatomy, Faculty of Medicine, Umm Al-Qura University, Makkah, Mecca 24382, Saudi Arabia; 6 Histology Department, Damietta Faculty of Medicine, Al-Azhar University, Damietta 34711, Egypt; 7 Department of Pathology, Qunfudah Faculty of Medicine, Umm-Al-Qura University Kingdom of Saudi Arabia, Makka 24382, Saudi Arabia; 8 Medical Laboratories Department, Faculty of Applied Medical Sciences, Umm Al-Qura University, Makkah 24382, Saudi Arabia; 9 Clinical Pharmacology Department, Faculty of Medicine, Mansoura University, Mansoura 35516, Egypt; 10 Department of physiology, Faculty of Medicine, Umm Al-Qura University, Makkah 24382, Saudi Arabia

**Keywords:** cyclosporine A, nephrotoxicity, ZW, apoptosis, autophagy, oxidative stress

## Abstract

Cyclosporine (CsA) is considered one of the main components of treatment protocols for organ transplantation owing to its immunosuppressive effect. However, its use is very restricted due to its nephrotoxic effect. ZW is an alkaline fluid rich in various trace elements and has a great ability to stimulate antioxidant processes. This study aimed to investigate the possible mitigating effect of ZW on CsA-induced nephrotoxicity and its underlying mechanisms. Forty rats were allocated into four groups (*n* = 10): a control group, ZW group, cyclosporine A group (injected subcutaneously (SC) with CsA (20 mg/kg/day)), and cyclosporine A+ Zamzam water group (administered CsA (SC) and ZW as their only drinking water (100 mL/cage/day) for 21 days). Exposure to CsA significantly (*p* < 0.001) increased the serum creatinine level, lipid peroxidation marker level (malondialdehyde; MDA), and the expression of apoptotic markers procaspase-8, caspase-8, caspase- 9, calpain, cytochrome c, caspas-3, P62, and mTOR in renal tissues. Meanwhile, it markedly decreased (*p*< 0.001) the autophagic markers (AMPK, ULK-I, ATag5, LC3, and Beclin-1), antiapoptotic Bcl-2, and antioxidant enzymes. Moreover, the administration of CsA caused histological alterations in renal tissues. ZW significantly (*p* < 0.001) reversed all the changes caused by CsA and conclusively achieved a positive outcome in restraining CsA-induced nephrotoxicity, as indicated by the restoration of the histological architecture, improvement of renal function, inhibition of apoptosis, and enhancement of autophagy via the AMPK/mTOR pathway.

## 1. Introduction 

Cyclosporine A (CsA), a natural cyclic peptide extracted from the fungus *Beauveria nivea*, is considered as one of the most important components of prophylaxis treatment protocols for organ transplantation and autoimmune diseases [1]. It inactivates T lymphocytes through the inhibition of the calcineurin enzyme via its attachment to the cytosolic protein cyclophilin [2]. Despite the use of cyclosporine as an immunosuppressive treatment for patients who have undergone kidney transplantation, it has severe side effects, one of which is nephrotoxicity [3]. 

Programmed cell death (apoptosis) has two pathways: (1) The first is the intrinsic (mitochondrial) pathway. This pathway is activated by several stimuli, including the Bcl-2 family and failure autophagy, which leads to the accumulation of misfolded proteins. The activation of the mitochondrial pathway activates caspase-9. (2) The second is the extrinsic pathway. This is activated through the death receptor, which eventually leads to the activation of caspase-8. This pathway can enhance the action of the mitochondrial pathway via the activation of the BH3 interacting-domain death agonist (Bid), a member of the Bcl-2 family [4,5]. Clear evidence of autophagosome formation is given by the conversion of the microtubule-associated protein light chain 3 (LC3 I) to (LC3 II), which takes place in autophagosomal membranes [6]. Currently, there is no clear explanation for the overlap between the apoptosis and autophagy pathways [7,8].

Previous studies in the literature have reported that the activation of autophagy has a crucial role in the alleviation of CsA-induced nephrotoxicity [9,10,11] and that the autophagy process is induced by endoplasmic reticulum stress [12]. Moreover, cyclosporine has been found to initiate invitro-programmed cell death in human proximal tubular epithelial cells [13] in a model of chronic nephropathy and to contribute to prolonging endoplasmic reticulum stress [14].

ZW is not chemically treated or chlorinated as is water pumped to the cities, and it is also free from biological growth. The experiment on ZW conducted at King Saud University in 2017 proved that it is unique in its characteristics, as it is free from any pathogens. The Saudi Arabian Standards Organization has also validated its trace element, anion, and cation contents [15]. Alfadul and Khan, [16] affirmed that ZW differs from distilled or boiled water due to its high amounts of trace elements including calcium (Ca), magnesium (Mg), sodium (Na), and chloride (Cl). Besides possessing numerous beneficial elements, it also has very low amounts of harmful components such as selenium, lead, cadmium, and arsenic. ZW has been examined in several studies for the therapeutic effects brought about by its healing character, which has also been attributed to the presence of trace elements [17]. Moreover, it has been reported that ZW showed anticancer activity through apoptosis induction in (HCT-116) cells in the human colon [18] and in lung cancer (A594) [19]. To the best of the authors’ knowledge, the potential ameliorative effect of ZW in response to CsA-induced nephrotoxicity has not yet been studied. Hence, this study was designed to assess the nephroprotective impact of ZW and its role in the crosstalk between autophagy and apoptosis in CsA-induced nephrotoxicity in male Wistar albino rats. 

## 2. Materials and Methods 

### 2.1. Animals

In this study, 40 rats weighing 200–250 g were obtained from the Medical Experimental Research Center in Mansoura University. These animals were kept in the Pharmacology Department of the Faculty of Veterinary Medicine in Mansoura University under standardized conditions at 24 ± 2 °C and a 12 h light–dark cycle. The rats were offered food and water ad libitum and were allowed to acclimate one week prior to the onset of the experiment. The Mansoura University Animal Care and Use Committee (MU-ACUC) endorsed the animal experiment (approval no. VM.R.22.10.20). 

### 2.2. Chemicals 

Cyclosporine (Sandimmune^®^, 50 mg/mL ampule) was purchased from Novartis (Istanbul, Turkey). ZW was brought from the Zamzam well located within the holy mosque in Makkah, Saudi Arabia. 

### 2.3. Experimental Design

The rats were divided into four groups (10 rats/group): a control group, in which rats received oral 0.9% saline at a rate of 0.3 mL/rat and tap water for 21 days; a ZW group, in which rats received ZW (100 mL/cage/day) as their only supply of drinking water for 21 days.; a CsA group, injected subcutaneously (SC) with CsA at 20 mg/kg/day and given oral 0.9% saline at a rate of 0.3 mL/rat for 21 days; and a CsA+ ZW group, wherein rats received an SC injection of cyclosporine at 20 mg/kg daily and were provided with ZW (100 mL/cage/day) as their only supply of drinking water for 21 days. The CsA dose used in this experiment was selected based on previous research published by Ateyya [20], El-Sheikh et al. [21], Abdel-latif et al. [22], and Mohamadin et al. [23], who reported that treatment with CsA at this dose causes severe nephrotoxicity. The dose of ZW used in this experiment was based on the study performed by Mraisel and Abu Ali, [24], who investigated its association in relation to kidney damage in rats. 

### 2.4. Specimen Collection 

The rats received anesthesia with sodium thiopental intraperitoneally at 50 mg/kg on day 21. The serum was obtained from blood harvested from the retroorbital venous plexus through a capillary tube. The rats were sacrificed via cervical dislocation. The kidneys were excised and washed with saline. The serum and portions of the kidneys were kept at −80 °C for further biochemical and molecular investigations. In addition, other parts of renal tissues were fixed in 10% formalin and then embedded in a paraffin block for histological and immunohistochemical assessment. The remaining kidney specimens were homogenized with a radioimmunoprecipitation assay buffer (RIPA buffer) completed with a cocktail of protease inhibitors, and the homogenate was then stored for 15 min at 4 °C. Following this, it was centrifuged at 15,000 g at 4 °C, and the resulting supernatant was stored at −80 °C for Western blotting assessment. 

### 2.5. Biochemical Examination 

#### 2.5.1. Renal Function Parameters

Blood urea nitrogen (BUN) and serum creatinine were assessed following the methods described by Kumar et al. [25] and Piéroni et al. [26], respectively. Additionally, creatinine clearance (Cr Cl) was estimated as follows:

$$Cr\;Cl(mL/min)=\frac{Urine\;volume\left(\frac{mL}{24\;h}\right)\times \;Urine\;creatininecon\;cnetration(\frac{mg}{dL})}{plasma\;creatinine(\frac{mg}{dL})}$$

#### 2.5.2. Assessment of Oxidative Stress Biomarkers 

Renal tissues were assessed for the lipid peroxidation marker malonaldehyde (MDA) with the use of commercial kits (Cat# No. MD 25-29) and the colorimetric method at 534 nm following the method of Mesbah et al. [27]. Furthermore, superoxide dismutase (SOD) activity was examined with colorimetry at 560 nm using commercial kits (Cat# No. SD25-21) as described by Xu et al. [28]. For the assessment of glutathione peroxidase (GPx), commercial kits (Cat# GP 2524) were used according to the instructions reported by Zhang et al. [29]. The activity of catalase (CAT) was investigated as described by Baureder et al. [30] (Cat# Ca 2517). All the kits were supplied by Biodiagnostics Co (Cairo, Egypt). 

### 2.6. Histopathological and Immunohistochemical Examination 

After fixation of the kidney in 10% formalin and its immersion in a paraffin block, 5 μm thick sections of the paraffin block were prepared for staining with hematoxylin and eosin (H&E) and Periodic acid–Schiff (PAS) stains. The kidney damage was evaluated and scored according to the following changes in the renal corpuscle: Bowman’s capsule and space widening, shrinkage of the glomeruli, nuclear pyknosis, and edema of the interstitial tissue and perivascular space. Another change observed was the alterations recorded in the renal tubules, which may come in the form of hyaline casts inside the renal tubular lumens. The scores were documented as published by Zahran et al. [31]. Five random fields for each slide were used for scoring. A score of 0 represented no damage, while a score of 1 indicated 10% damage, that of 2 indicated 11–25% damage, a score of 3 represented 26–45% damage, a score of 4 represented 46–75% damage, and a score of 5 indicated more than 76% damage. For the immunohistochemical examination of the renal tissues, 5 μm thick sections were deparaffined using xylene with the descending scale of ethanol for dehydration. To retrieve the antigen, sections of the renal tissues were immersed inside a hot buffer (citrate) for 10 min, and then 0.3% H_2_O_2_ was applied. Thereafter, the sections were incubated with the primary antibodies of LC3 (Cat# GB13431 1:300), P62 (Cat# A19700 1:200), Beclin 1 (Cat# GB11228 1:500), Caspase 3 (Cat# GB11532 1:500), Bax (Cat# A12009 1:100), and Bcl-2 (Cat# Clone 124 1:200). Following this, the enzyme horseradish peroxidase (HRP) (Dako C., Denmark) and secondary antibody (goat antirabbit, Cat# K205587, EN vision) were incubated at 25 °C for half an hour. Sections were mounted with 3,3′ diaminobenzidine and then counter-stained with hematoxylin. The slides were examined by histopathologists at the Department of Pathology, Faculty of Veterinary Medicine, Mansoura University, Egypt. 

### 2.7. Western Blotting of Autophagy-Activating Kinase ULK1, Atg5, Procaspase-8, Caspase-8, Caspase-9, Calpain, and Cytochrome c 

To evaluate the protein content of renal tissues, the Western blotting technique was employed according to the process described by Abdel-Naim et al. [32]. A total of 30 g of tissue was lysed before the assessment of the supernatant’s protein contents; a phosphate inhibitor cocktail was utilized (Cat# P0001.SIGMA. ALDRICH, Street Louis, MO, USA) in a cold RIPA lysis buffer. The protein contents of the samples were subjected to polyacrylamide gel electrophoresis and then transferred onto polyvinylidene difluoride membranes (Bio-Rad Co, Hercules, CA, USA). Thereafter, the membranes were blocked with five-percent milk in Tris-buffered saline comprising 0.1% Tween 20 solution for one hour before being incubated overnight at 4 °C with primary antibodies anti-AMPK (Cat# 16,868,224 1:500), anti-mTOR (Cat# 2972 1:1000), anti-ATAG-5 (Cat# NB 110.53818 1:500), anti-Caspase-3 (Cat# 9662 1:1000), anti-procaspase-8 (Cat# ab108333 1:1000), anti-caspase-8 (Cat# sc-56070 1:200), anti-caspase-9 (Cat# sc-73548 1:200), cytochrome c (Cat# 4272 1:1000), Calpain 1 (Cat# sc-271313 1:500), and anti-phospho-ulk1 antibody (Cat# ser-757 1:1000). The membranes were then cleared and incubated for one hour at room temperature with the HRP-conjugated secondary antibody (Cat # H10307 1:2000) (Goat anti-rabbit IgG- HRP-1 mg Goat mab -Novus Biologicals). The chemiluminescent substrate (BIO-RAD Cat# 170~5060) was then applied to the blot with solution A and solution B, and then another washing with Tris-phosphate saline was performed. In order to examine the target proteins’ banding intensity with respect to the housekeeping protein B actin (cat# ab115777 1:200), the CHEMI-DOC MP imager for chemiluminescent records was used.

### 2.8. Analysis of Apoptosis with Flow Cytometry

The primary proximal tubular epithelial cells (PTECs) from different experimental groups were isolated according to the previous method performed by Liu et al. [33]. The cells were maintained in K1 culture medium DMEM/F12 + GlutaMax (Invitrogen GmbH); supplemented with 5% FBS (PAN-Biotech GmbH; Aidenbach, Germany), 1% penicillin/streptomycin, insulin (5 μg/mL), hydrocortisone (1.8 μg/mL), transferrin (5 μg/mL), sodium selenite (173 ng/mL), murine epidermal growth factor (25 ng/mL), and triiodothyronine (6.5 ng/mL); and then seeded into collagen R-coated (20 μg/mL) 6-well culture plates (TPP AG, Trasadingen, Switzerland) at a density of approximately 1 × 105 cells per well. The cells were incubated with 5% CO_2_ at 37 °C, in a similar fashion to that conducted with the rat cell line NRK52E, for 24 h. The cells were collected in free trypsin- EDTA, centrifuged for 5 min at 1000 rpm, and then washed with PBS. After being resuspended in the binding buffer, the cells were treated with the use of Annexin V−FITIC and PI apoptosis detection kits (KEYGen BioTech, Nanjing, China) for 15 min and were subsequently analyzed with flow cytometry (Becton Dickinson). Channel FL1 was used to detect Annexin V−FITIC green fluorescence for the test, and channel PI was used to detect red fluorescence (FL3). The Accuri C6 package was used to calculate the Annexin V−FITIC and PI-stained cells.

### 2.9. RT-PCR Assessment

Total RNA was isolated from the tissue homogenate of the different groups using Direct-Zol RNA Miniprep plus (Cat# R2070. ZYMO research corporation, Irvine, CA USA). A Beckmann Daul spectrophotometer (USA) was used for quantitative and qualitative determination. Thermos Fisher Scientific (Waltham, MA, USA) provided the SuperScript IV One-Step RT-PCR kit (Cat# 12594100) for the determination of the reverse transcriptase and PCR of the isolated RNA. Using a 96-well Step One instrument (Applied biosystems, Foster City, CA, USA), thermal profiling was performed as follows: reverse transcription for 10 min at 45 °C; RT inactivation for 2 min at 98 °C; and initial denaturation for 40 cycles of 10 s at 98 °C, 10 s at 55 °C, and 30 s at 72 °C. Real-time PCR results for the housekeeping and target genes are expressed in cycle threshold. The 2-∆∆Ct method was used to determine the mean critical threshold expression value of the GAPDH housekeeping gene and the target gene in order to minimize the expression variation. Primer sequences for the AMP-activated protein kinase (AMPK), mammalian target of rapamycin (mTOR), and glyceraldehyde3. phosphate (GAPDH) genes are listed in Table 1. 

### 2.10. Analysis of Zamzam Water

A total of 300 mL of ZW was obtained from Al Haram pipes using a 500 mL glass bottle. The analysis was completed in 24 h using atomic absorption spectrophotometry measurements from the SETE Saudia Building Quality for life (ALFATAH WATER AND POWER) company, Jeddah, Saudi Arabia.

### 2.11. Statistical Analysis

The data were analyzed using the Graph Pad Prism program version 8.0 (La Jolla, CA, USA). The values of different groups are presented as mean ± SD, and a comparison of the different groups was carried out with one-way ANOVA, followed by the Tukey–Kramer post hoc test. *p* values less than 0.05 were regarded as significant.

## 3. Results 

### 3.1. Effect of Zamzam Water on CsA A-Induced Renal Impairment

The rats that received CsA revealed remarkable renal impairment as compared to the control group, which was evidenced by the significant elevation (*p* < 0.001) of the serum level of creatinine and BUN, with a marked reduction (*p* < 0.001) in creatinine clearance. Interestingly, drinking ZW with CsA injection significantly (*p* < 0.001) improved renal function by decreasing creatinine and BUN serum levels as well as increasing creatinine clearance relative to the CsA group (Table 2); there was no significant difference between the control group and ZW group. 

### 3.2. Effect of Zamzam Water against CsA-Induced Oxidative Stress 

Rats inoculated with CsA exhibited a significant (*p* < 0.001) elevation of the lipid peroxidation marker (MDA) level and a reduction in the activities of antioxidant enzymes SOD, CAT, and GPx in renal tissues as compared to control rats. ZW, with its powerful antioxidant character, markedly (*p* < 0.001) decreased the homogenate level of MDA and enhanced the activities of SOD, CAT, and GPx in comparison with the CsA group (Table 3). There were no significant differences between the control group and ZW group despite the increase in antioxidative stress markers in the ZW group.

### 3.3. Ameliorative Impact of Zamzam Water on Renal Morphology 

**Figure 1:**
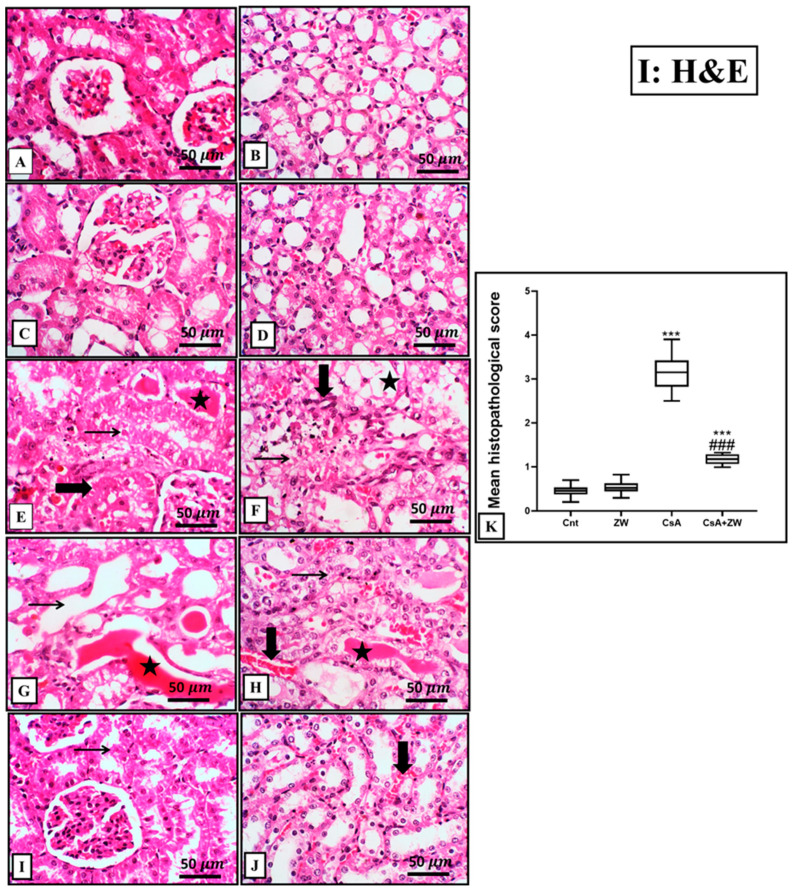
Representative photomicrograph of renal tissues from the control and experimental groups stained with H&E. (**A**,**C**) The control and ZW groups show cortical glomeruli and tubules with normal architecture. (**B**,**D**) The control and ZW groups show medullary tubules with normal histology. (**E**) The CsA group shows a diffuse loss of cellular detail with hypereosinophilic cytoplasm and nuclear pyknosis, karyorrhexis, and karyolysis (necrosis) (thin arrow), along with swollen, vacuolated cytoplasm with faded nuclei beside intraluminal eosinophilic globules admixed with sloughed tubular epithelial cells, necrotic debris (granular casts) (star), and proliferative glomerular capillaries (thick arrow). (**F**) CsA medullary tubules underwent diffuse medullary tubular necrosis (thin arrow) and vacuolation (star) with focal interstitial fibrosis (thick arrow). (**G**) The CsA group shows diffuse necrotic cortical tubules with a large intraluminal hyaline cast. (**H**) Medullary tubular sloughing (thin arrow) and intraluminal hyaline cast (star) with interstitial congestion (thick arrow). (**I**) The CsA + ZW group displays occasional individual tubular necrosis (thin arrow). (**J**) The CsA + ZW group exhibits diffuse minimal-to-mild interstitial congestion (thick arrow). (**K**) Histogram of the percentage area of the histological score. *** *p* < 0.001 vs. *control*; *^###^ p* < 0.001 vs. CsA. Image magnification = 400×; scale bar = 50 µm.

**Figure 2:**
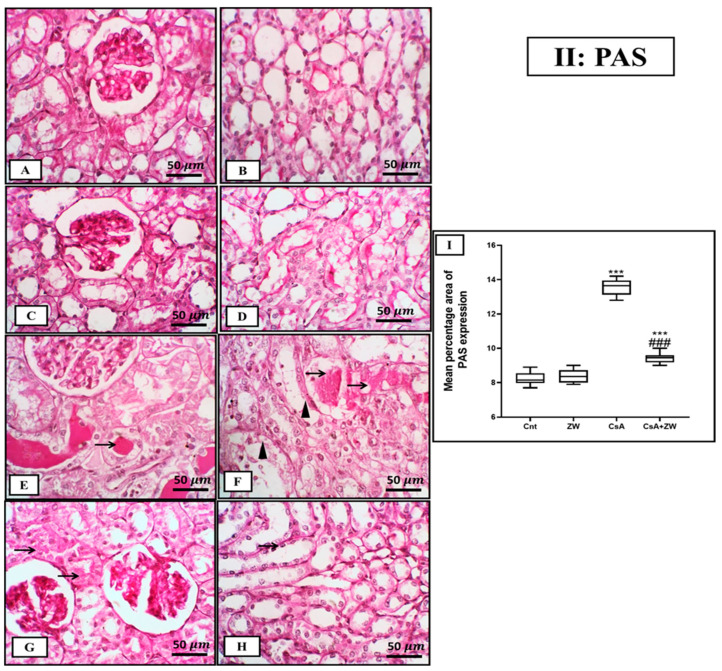
Photomicrograph of PAS expression in kidneys from control and experimental rats. (**A**–**D**) The control and ZW groups show PAS highlights of the glomerular basement membrane and tubular epithelium of the cortical and medullary region. (**E**,**F**) The CsA group shows PAS-positive staining in the dilated cortical and medullary tubules (arrowheads) with intraluminal proteinaceous globules (thin arrows). (**G**,**H**) The CsA + ZW group shows focal intracytoplasmic-positive PAS in the tubular epithelium of the cortex and the positive staining of the tubular basement membrane of medullary tubules (thin arrows). (**I**) Histogram of the percentage area of PAS expression. *** *p* < 0.001 vs. *control*; *^###^ p* < 0.001 vs. CsA. Image magnification = 400×, bar = 50 µm.

A histopathological investigation of the kidneys of the control and ZW groups showed normal tubules and glomeruli (Figure 1A–D). On the contrary, treatment with CsA revealed significant (*p* < 0.001) alternations in the renal architecture in the form of diffuse necrosis in the cortical cells, with the presence of hypereosinophilic cytoplasm and nuclear pyknosis with large intraluminal sloughed tubular epithelial cells and necrotic debris (granular casts) as well as proliferative glomerular capillaries (Figure 1E,G). Furthermore, medullary tubules showed diffuse medullary tubular necrosis and vacuolation with focal interstitial fibrosis (Figure 1F,H). Drinking ZW was associated with a marked histological improvement in the form of occasional individual tubular necrosis with diffuse minimal-to-mild tubular interstitial congestion (Figure 1I,J). Additionally, CsA administration was associated with marked glycogen deposition in the form of PAS-positive staining in dilated cortical and medullary tubules with intraluminal proteinaceous globules (Figure 2E,F). In contrast, the CsA+ ZW group showed a significant decrease (*p* < 0.001) in glycogen deposition in the form of focal intracytoplasmic-positive PAS in the tubular epithelium of the cortex and a positive staining of the tubular basement membrane of medullary tubules (Figure 2G,H). This improvement in the morphological structures of the renal glomerulus and tubules explains the ameliorative effect of ZW on renal function.

### 3.4. Effect of Zamzam Water against CsA-Induced Renal Tubular Apoptosis 

**Figure 3:**
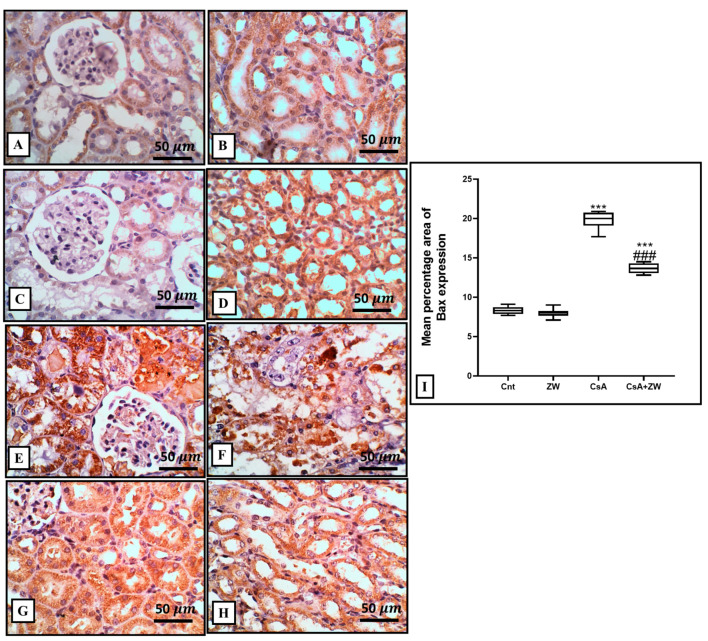
Bax immunohistochemistry of the kidney. (**A**–**D**) The control and ZW groups show faint cytoplasmic Bax expression in normal cortical and medullary tubules. (**E**,**F**) The CsA group shows diffuse severe and strong Bax expression in the cytoplasm of necrotic, sloughed tubular cells and vacuolated cells. (**G**,**H**) The CsA + ZW group shows diffuse moderate Bax expression in cortical and medullary tubules. (**I**) Histogram of the percentage area of the immunostaining of renal Bax. *** *p* <0.001 vs. *control*; *^###^ p* < 0.001vs. CsA. Image magnification = 400×; scale bar = 50 µm.

**Figure 4:**
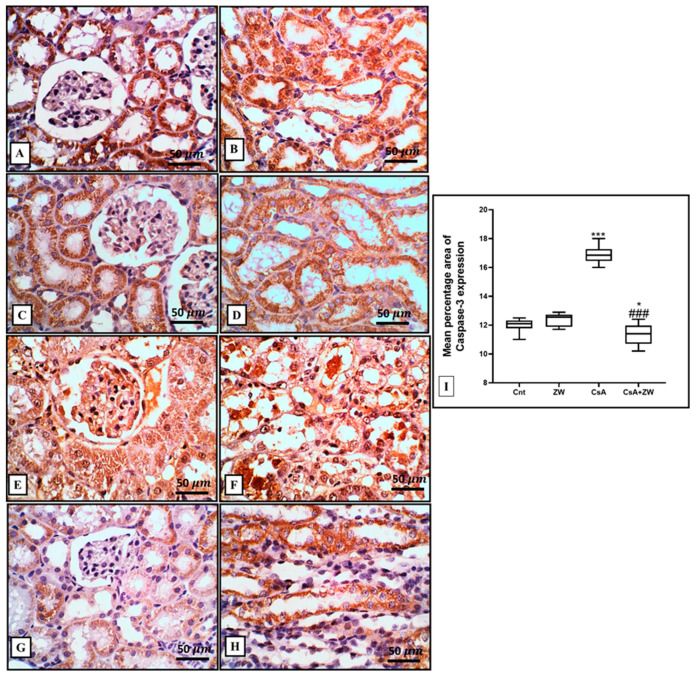
Immunohistochemical analysis of caspase-3 expression and localization in the kidney. (**A**–**D**) The control and ZW groups show cytoplasmic expression in the normal cortical and medullary tubular epithelium. (**E**,**F**) The CsA group shows diffuse, strong expression in the glomerulus, in the cytoplasm of necrotic and vacuolated cortical and medullary tubular cells, and in the cytoplasm of vacuolated and intraluminal cells. (**G**,**H**) The CsA + ZW group shows moderate cortical and medullary tubular expression. (**I**) Kidney caspase 3 histogram. *** *p* < 0.001, and * *p* < 0.05 vs. *control*; *^###^ p* < 0.001 vs. CsA. Image magnification = 400×; scale bar = 50 µm.

**Figure 5:**
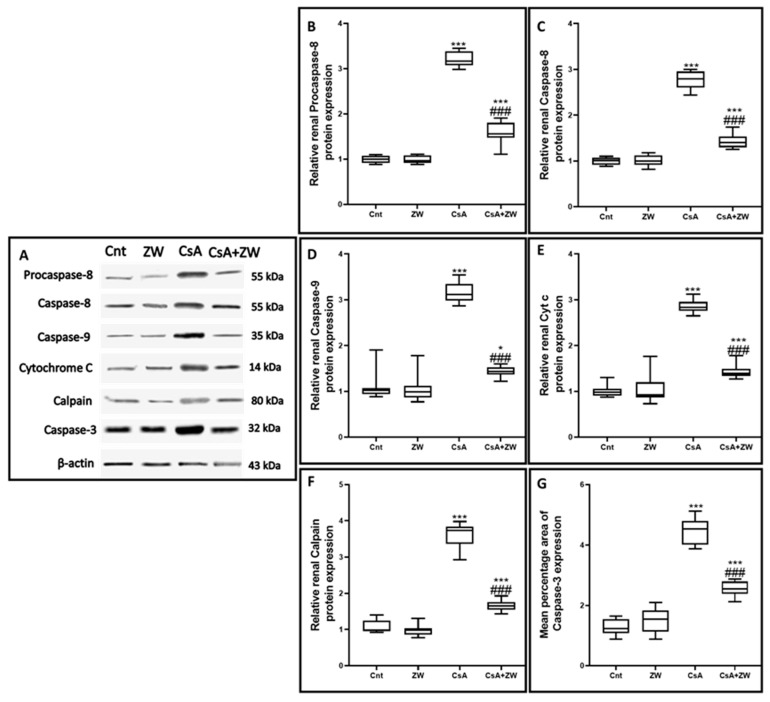
The effect of ZW on the expression of the proteins (**A**,**B**) procaspase-8, (**A**,**C**) caspase-8, (**A**,**D**) capase-9, (**A**,**E**) cytochrome C, (**A**,**F**) calpain, and (**A**,**G**) caspase-3 according to Western blotting assay. *** *p* < 0.001, and * *p* < 0.05 vs. *control*; *^###^ p* < 0.001 vs. CsA.

**Figure 6:**
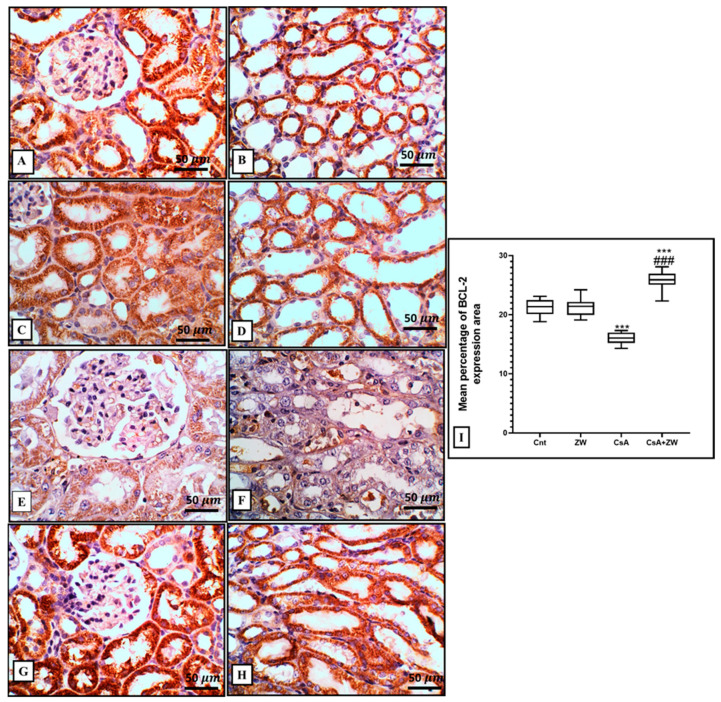
Representative Bcl2-immunohistochemistry of the kidney. (**A**–**D**) The control and ZW groups show diffuse strong expression in the normal cortical and medullary tubular epithelium. (**E**,**F**) The CsA group shows diffuse moderate expression in necrotic, sloughed tubular cells and in the cytoplasm of vacuolated and intraluminal cells. (**G**,**H**) The CsA + ZW group shows diffuse strong Bcl-2 expression in the cortical and medullary tubules. (**I**) Histogram of the percentage area of immunostaining of renal Bcl-2, *** *p* < 0.001 vs. *control; ^###^ p* < 0.001 vs. CsA. Image magnification = 400×; bar = 50 µm.

**Figure 7:**
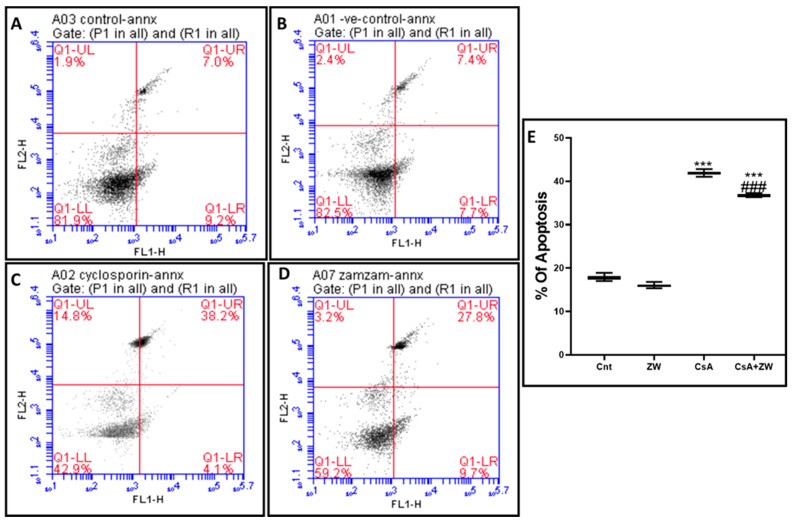
Flow cytometry analysis of renal tubular cells stained with the apoptotic marker Annexin V as an indifferent experimental group. (**A**) Control group, (**B**) ZW group, (**C**) CsA group, and (**D**) CsA + ZW group. (**E**) Histogram of apoptotic cells. *** *p* < 0.001 vs. *control*; *^###^ p* < 0.001 vs. CsA.

The subcutaneous injection of CsA induced a substantial (*p*< 0.001) rise in the immunoexpression of the proapoptotic protein Bax (Figure 3E,F) and the number of renal tubular apoptotic caspase-3 cells (Figure 4E,F). Additionally, it increased the protein expression of the apoptotic proteins caspase-3, procaspase-8, caspase-8, caspase-9, cytochrome c, and calpain, as indicated by the Western blot assay (Figure 5). It also significantly (*p* < 0.001) decreased the immunoexpression of Bcl-2 (Figure 6E,F) in comparison to the control group. Interestingly, the concomitant administration of ZW and CsA significantly (*p* < 0.001) antagonized their apoptotic effect on renal tubular epithelial cells by decreasing the immunoexpression of Bax (Figure 3G,H) and the Western blotting protein expression of caspase-3, procaspase-8, caspase-8, caspase-9, cytochrome c, and calpain (Figure 5). Moreover, ZW upregulated the immunoexpression of Bcl-2 (Figure 6G,H). With regard to the flow cytometric examination of in vitro Annexin-V-labeled renal tubular apoptotic cells, CsA significantly (*p* < 0.001) increased the level of the labeled apoptotic cells, which counterweighed the control group (Figure 7C). Interestingly, ZW administration significantly (*p* < 0.001) decreased the count of apoptotic cells in comparison to the CsA group. ZW exhibited a cytoprotective function in response to CsA-induced renal tubular apoptosis by inhibiting the extrinsic apoptotic pathway proteins procaspase-8 and caspase-8 as well as the intrinsic pathway proapoptotic protein Bax, mitochondrial membrane protein cytochrome c, and caspase-9 protein. Subsequently, there was a decrease in the percentage of cleaved caspase-3 apoptotic cells with the activation of the antiapoptotic protein Bcl-2.

### 3.5. Effect of Zamzam Water on Autophagic Magnitude in the Kidney Epithelial Cells of Rats with CsA-Induced Nephrotoxicity 

**Figure 8:**
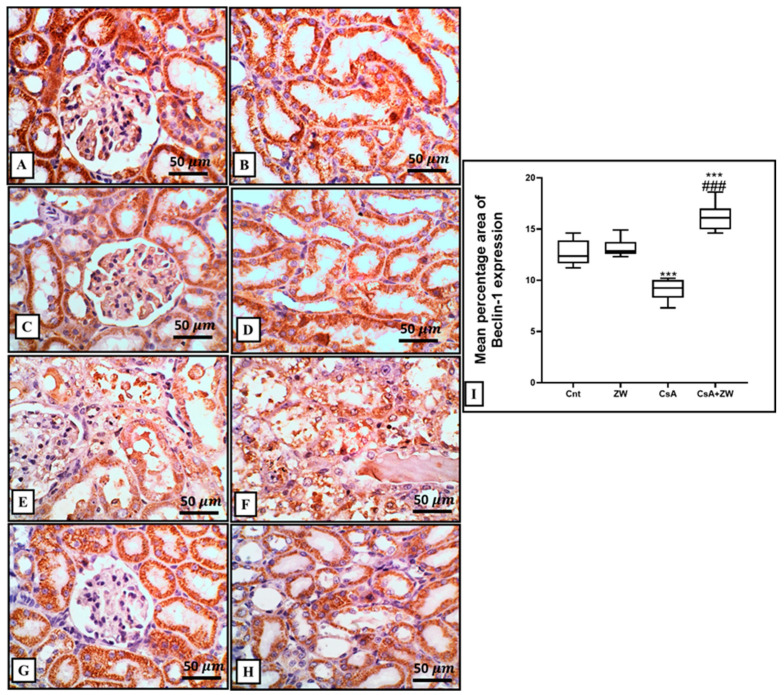
Immunohistochemical investigation of beclin-1 expression and localization in the kidney. (**A**–**D**) The control and ZW groups show strong cytoplasmic expression in cortical tubules, with mild glomerular expression and diffuse expression in the cytoplasm of medullary tubules. (**E**,**F**) The CsA group shows diffuse, faint expression in the cytoplasm of necrotic and vacuolated cortical and medullary tubular cells and in the cytoplasm of vacuolated and intraluminal cells, with faint minimal expression in the glomerulus. (**G**,**H**) The CsA + ZW group shows diffuse, high cortical and medullary tubular expression, with little glomerular expression. (**I**) Renal Becline_1 histogram percentage area. *** *p* < 0.001 vs. *control*; *^###^ p* < 0.001 vs. CsA. Image magnification = 400×; scale bar = 50 µm.

**Figure 9:**
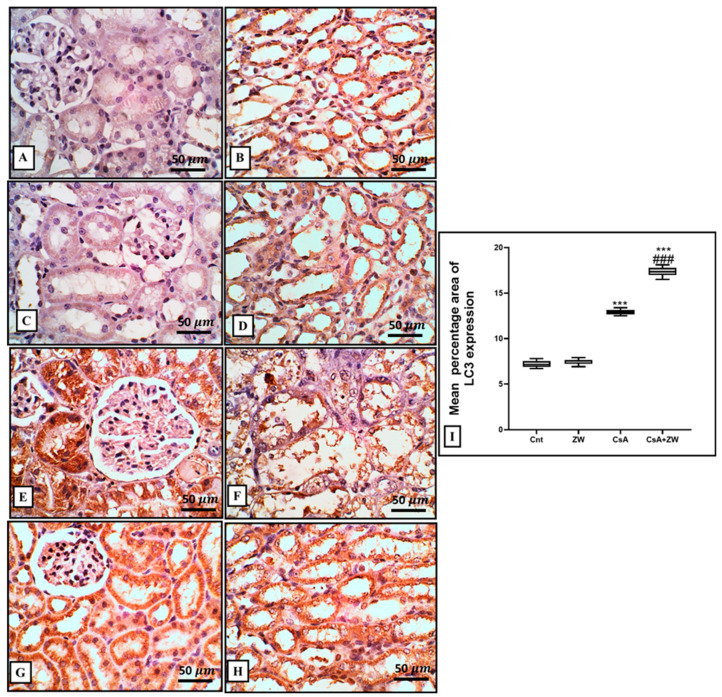
Representative immunohistochemical assessment of LC3 expression in the kidney. (**A**–**D**) The control and ZW groups show none-to-few faint expressions in cortical tubules without glomerular expression and diffuse expression in the cytoplasm of medullary tubules. (**E**,**F**) The CsA group shows diffuse strong expression in the cytoplasm of necrotic, sloughed, and vacuolated cortical cells, with few glomerular expressions and a lower expression in medullary tubular cells (intraluminal sloughed cells and intraluminal cast). (**G**,**H**) The CsA+ ZW group shows diffuse cortical and medullary tubular expression with low glomerular expression. (**I**) Histogram of the percentage area of the immunostaining of renal LC3. *** *p* < 0.001 vs. *control*; *^###^ p* < 0.001 vs. CsA. Image magnification = 400×; scale bar = 50 µm.

**Figure 10:**
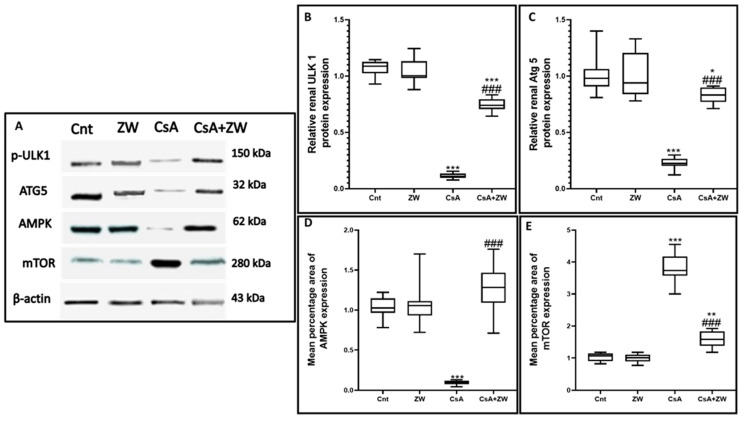
Influence of ZW on the protein expression of (**A**,**B**) P UlK-1, (**A**,**C**) Atg5, (**A**,**D**) AMPK, and (**A**,**E**) mTOR proteins according to Western blotting assay. *** *p* < 0.001, ** *p* < 0.001, and * *p* < 0.05 vs. *control*; *^###^ p* < 0.001vs. CsA.

**Figure 11:**
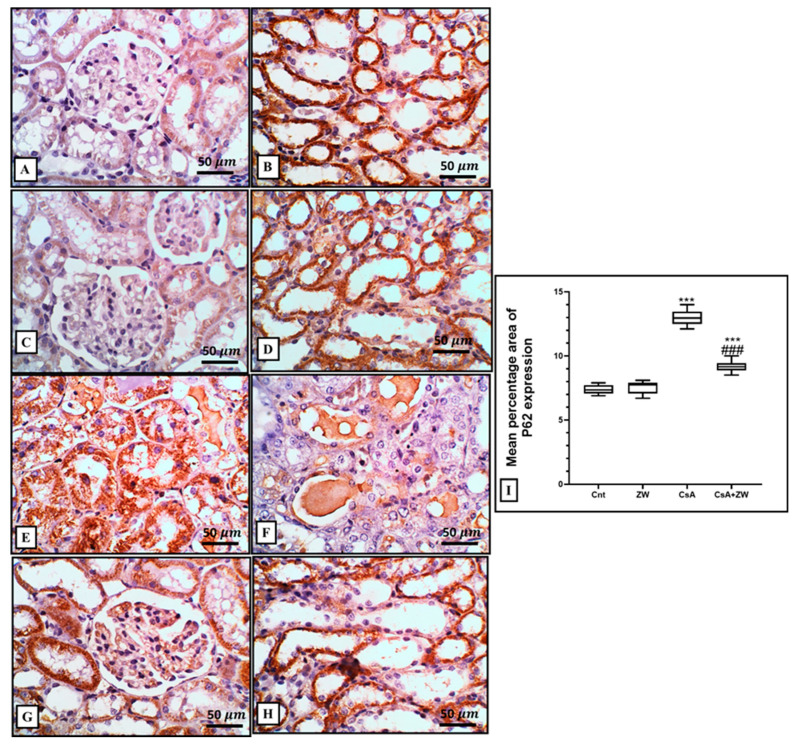
Representative immunohistochemical analysis of P62 expression and localization in the kidney. (**A**–**D**) The control and ZW groups show few faint cytoplasmic expressions in the cortical tubules without glomerular expression and diffuse expression in the cytoplasm of the medullary tubules. (**E**,**F**) The CsA group shows diffuse strong expression in the cytoplasm of the necrotic and vacuolated cortical, with lower expression in the medullary tubular cells (intraluminal sloughed cells and intraluminal cast). (**G**,**H**) The CsA + ZW group shows diffuse cortical and medullary tubular expression with glomerular expression. (**I**) Histogram of the percentage area of the immunostaining of renal P62. *** *p* < 0.001 vs. *control*; *^###^ p* < 0.001 vs. CsA. Image magnification = 400×; scale bar = 50 µm.

In comparison to the control group, CsA administration downregulated autophagic renal tubular epithelial cell death by significantly (*p* < 0.001) decreasing the immunoexpression of the autophagy regulator protein Beclin-1 and the autophagosome membrane protein LC3 II (Figure 8E,F and Figure 9E,F), with the downregulation of the protein level of autophagy-related protein ATg5. This was elucidated with Western blot analysis (Figure 10C) and the upregulation of the immunoexpression of P62 (Figure 11E,F), which is considered to be an indicator of the inhibition of the autophagic process. On the other hand, ZW counteracted the negative effect of CsA on the autophagy flux by the significant (*p* < 0.001) upregulation of Beclin-1 and LC3 II immunoexpression and the protein level of Atg5, which was concomitant with the downregulation of P62 immunoexpression in relation to the CsA group. This indicates that ZW improved cellular homeostasis by promoting autophagic renal tubular epithelial cell death and enhancing cellular survival in response to cellular apoptosis shift.

### 3.6. Effect of Zamzam Water on the AMPK/mTOR Pathway 

**Figure 12:**
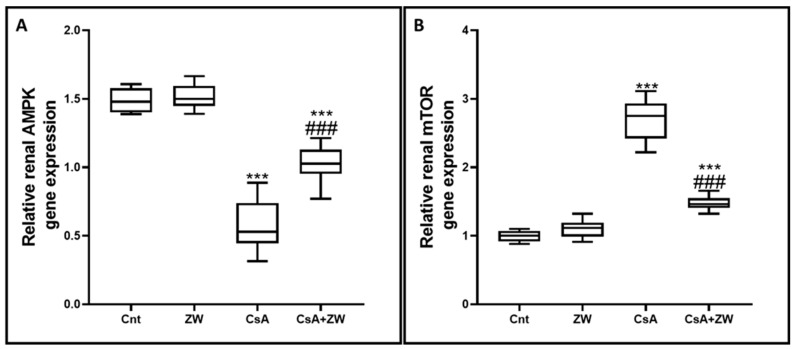
The effect of ZW treatment on the expression of the (**A**) AMPK and (**B**) mTOR genes in various groups. *** *p* < 0.001 vs. *control*; *^###^ p* < 0.001 vs. CsA.

In order to elucidate the positive effect of ZW on the autophagic process, its effect on the AMPK/mTOR pathway, which is considered to be the main regulatory pathway in the autophagy process, was examined. The subcutaneous injection of CsA significantly (*p* < 0.001) inhibited the AMPK–ULK-1 interaction by downregulating the phosphorylation of AMPK (Figure 10D and Figure 12A) and ULK-1 (Figure 10B) at the level of gene expression for AMPK and at the protein level for AMPK and ULK-1. This is considered to be a positive pathway for the induction of the autophagic process during CsA-induced nephrotoxicity. Furthermore, the phosphorylation of mTOR (Figure 10E and Figure 12B), a negative regulator of autophagy, was upregulated in CsA group as compared to the control group. Interestingly, drinking of ZW significantly (*p* < 0.001) upregulated AMPK at the mRNA and protein levels while upregulating ULK-1 at the protein level; mTOR was also downregulated at the mRNA and protein levels in CsA+ZW group in comparison with the CsA group. The regulatory effect of ZW on the AMPK/mTOR pathway indicated its positive effect on the autophagy process.

## 4. Discussion 

Cyclosporine A administration in organ transplantation is mandatory owing to its immunosuppressive properties, but its prolonged usage causes many side effects, one of which is oxidative-stress-induced nephrotoxicity [34,35,36]. It has been reported that antioxidant intake positively protects individuals against oxidative-insult-induced nephrotoxicity [34]. The antioxidant power of ZW is highly regarded for its alkaline ionized nature [20,37,38]. Analyses of ZW have revealed its high amounts of beneficial trace elements such as zinc, Mg, and selenium, which give ZW its antioxidant and antiapoptotic character [39,40]. Therefore, this study investigated the possible protective effect of ZW against CsA-induced nephrotoxicity along with its underlying mechanisms. From our findings, we can conclude that ZW improves renal impairment induced by CsA administration by decreasing creatinine and BUN levels and elevating creatinine clearance. Moreover, drinking ZW combats CsA-induced oxidative stress by decreasing MDA, a lipid oxidation marker, and enhancing the activities of the antioxidant enzymes SOD, GPx, and CAT, resulting in a marked improvement in the renal architecture. Additionally, ZW eliminates renal tubular epithelial cell apoptosis via a decrease in the apoptotic markers procaspase-8, caspase-8, case-9, caspase-3, cytochrome c, and BAX along with the augmentation of antiapoptotic Bcl-2 immunoexpression as well as the upregulation of the autophagy markers Beclin-1, LC3, and Atg5 and the downregulation of P62 immunoexpression. 

Our findings revealed that CsA administration significantly (*p* < 0.001) deteriorated the renal function markers creatinine and BUN, which is in agreement with the studies by Haleagrahara et al. [41] and Helmy et al. [42]. This can be regarded as the reduction of the glomerular filtration rate or as tubular damage resulting from cyclosporine administration [43,44]. This renal impairment appeared as diffuse cortical cell necrosis with large intraluminal sloughed tubular epithelial cells and necrotic debris (granular casts). These histological alternations were in line with previous studies by Yoon [45] and Lee, [46]. 

The current study indicated that the subcutaneous injection of CsA significantly (*p* < 0.001) downregulated the antioxidant enzymes CAT, GPx, and SOD and enhanced MAD as a lipid peroxidation marker. These results are consistent with previous work performed by Capasso et al. [47]. CsA-induced oxidative stress due to the overproduction of oxygen free radicals consumes the protective endogenous antioxidant enzymes, resulting in cellular lipid, DNA, and protein damage [48,49]. MDA elevation results in cellular membrane damage, mainly in the lipid membrane [50]. CsA-induced histological alternation can be explained by the accumulation of reactive oxygen species (ROS), which negatively affects the glomerular endothelium [51]. Hyaline casts can be explained by lipid peroxidation, which disrupts cellular membranes and by the outpouring of cytoplasmic contents into the tubular lumen [52]. 

ZW drinking water significantly (*p* < 0.001) improved renal function, downregulated the serum levels of creatinine and BUN, and increased creatinine clearance. These results are in accordance with the findings of El Maleky et al. [53], who stated that drinking ZW with an oral gliclazide hypoglycemic drug markedly normalizes the serum BUN, creatinine, and albumin in STZ-induced nephropathy. Furthermore, Abdullah et al. [54] stated that drinking ZW, which is a good example of alkaline water, exhibited remarkable improvements in metabolic acidosis concomitant with chronic renal failure and hemodialysis. This renoprotective effect of ZW can be explained by its antioxidant power. Considering the higher exposure of renal tubular cells to oxidative stress due to their oxygen consumption [55,56], drinking ZW was shown to significantly (*p* < 0.001) elevate the homogenate endogenous antioxidant enzymes CAT, GPx, and SOD, while additionally reducing the level of the lipid peroxidation marker MDA. This result is in line with previous research conducted by Abdullah et al. [53], Satta et al. [57], and Bamosa et al. [58]. The antioxidant effect of ZW is highly regarded for its ionized nature, and its analysis revealed its high amounts of Zn, Mn, and Se (Table 4), which have high antioxidant power and a remarkable elevation in their antioxidant enzyme levels [53]. The correction of renal functions by ZW via ROS scavenging also explains the improvement in the renal histological architecture. These findings align with the study by Saif et al. [38], who discussed the effect of ZW in response to carbon-tetrachloride-induced liver-architected modifications, and with the findings of El Maleky et al. [53], who reported on the protective effect of ZW in renal histology in STZ-induced diabetic nephropathy.

The pathogenesis of CsA-induced nephrotoxicity is considered to involve the activation of several apoptotic cascades in renal tubular epithelial cells [59]. Three pathways are involved in the pathogenesis of the apoptotic process: the death receptor activation extrinsic pathway, mitochondrial stress intrinsic pathway, and ER stress pathway [60]. There is a substantial link between the mitochondrial pathway and oxidative stress-induced apoptosis [61]. In this study, the Western blotting assay showed that ZW significantly (*p* < 0.001) increased the protein expression of procaspase-8, caspase-8, calpain, caspase-3, and cytochrome c and that it increased the percentage of Annexin-V-labeled apoptotic renal cells. Several pathways are associated with apoptotic cell death via CsA administration, including oxidative stress via the overexpression of the proapoptotic p53 protein resulting from ROS overproduction, as reported by Koppelstaetter et al. [62]. These findings are in agreement with other works by Tu et al. [63] and Øzbay et al. [64], who reported the DNA fragmentation of CsA and its implication in the pathogenesis of chronic organ diseases. Interestingly, drinking ZW significantly antagonized the apoptotic effect of CsA against renal tubular epithelial cells by decreasing caspase-3 expression and improving its effect on the antiapoptotic protein Bcl-2. These findings are in line with those of El Maleky et al. [53], who proved the antiapoptotic effect of the combination of ZW with gliclazide by exploring their capacity to decrease the immunoexpression of renal tubular epithelial cells stained with caspase-3 in STZ-induced nephropathy. We thus conclude that the antiapoptotic power of ZW can be regarded as its antioxidant property. 

Several works have explored the effect of oxidative stress on the regulation of autophagy [65]. Several studies have also discussed the protective role of the autophagy process, which serves as a cellular defense mechanism against CsA-induced nephrotoxicity [66]. Autophagy maintains cellular homeostasis by balancing cellular component degradation and production, thus delaying the apoptotic cellular cells [67]. The results of the present work revealed that CsA significantly (*p* < 0.001) downregulated the protein expression of the autophagic protein Atg5 and the immunoexpression of the autophagic regulator Beclin-1. It also induced the autophagosome membrane formation of the LC3-II puncta with the upregulation of P62, which is considered to be one of the autophagy-associated proteins whose accumulation is related to autophagy inhibition. However, our results are in disagreement with those of Pallet et al. [10], who demonstrated the upregulating effect of CsA on human-cultured renal tubular epithelial cells through an increase in LC3-II protein expression and autophagosome formation with electron microscopic examination. However, our results are consistent with those of Inoue et al. [68] and Jiang et al. [69], who reported that the autophagy process was upregulated in the initial phases of cisplatin-induced severe kidney pain as a protective mechanism against tubular epithelial cell apoptosis. The works of Li et al. [70] and Ozkok et al. [71] also confirm the previous explanation with their investigations, which showed that the treatment of NRK-52E cells with 20 mmol/L cisplatin markedly upregulated LC3II in the first 6 h of the experiment; however, after 24 h, LC3II markedly decreased to basal level. On the other hand, Zamzam water exerted an upregulating effect on the autophagy process by upregulating the protein expression of Atg5 and the immunoexpression of LC3 and beclin-1 and by decreasing the immunoexpression of P62. This increased the autophagic flux in comparison to the CsA group and indicated that drinking ZW prolongs the procaspase lag period and the subsequent inhibition of renal tubular epithelial cell apoptosis, thus mitigating CsA-induced nephrotoxicity. In the current research, at the mRNA level and with the determination of protein expression using Western blotting, cyclosporine A was shown to significantly (*p* < 0.001) decrease the phosphorylation of AMPK and increase mTOR phosphorylation. Moreover, Western blotting revealed the decreased protein expression of ULK-1, an autophagy initiator. The downregulating effect of CsA on the autophagy process was most probably due to its impact on the AMPK/mTOR pathway. This result is in disagreement with the findings reported by Park et al. [72], who reported that the intraperitoneal injection of cyclosporine at a dose of 50 mg/kg increased the AMPK level in a rat’s hippocampus 5 h after injection. This can be explained as CsA’s upregulating effect on the autophagy process at the acute stage. Several studies have demonstrated that the phosphorylation of AMPK potentiates the autophagy process by activating the ULK-1 autophagy initiator kinase under starvation circumstances. In contrast, mTOR phosphorylation inhibits autophagy processes under normal conditions by interfering with the interaction between AMPK and ULK-1 [73]. Recent research has demonstrated that the activation of the AMPK signal has an ameliorative effect in response to acute kidney injury [74]. Drinking ZW was shown to activate the autophagy process in response to CsA-induced renal tubular apoptosis through its modulation of the AMPK/mTOR pathway. Autophagy-apoptosis cellular death is considered to be complex pathway [7,8]. There are three different types of interactions between autophagy and apoptosis which illustrate their crosstalk: First, autophagy collaborates with apoptosis; second, autophagic cellular death antagonizes apoptotic cellular death; third, autophagy enables apoptotic cellular death. The findings of this research showed that CsA injection could upregulate proapoptotic Bax protein immunoexpression and downregulate antiapoptotic Bcl-2 protein immunoexpression, which resulted in the DNA fragmentation of the renal tubular epithelial cell and its subsequent apoptosis. The transfer of Bcl-2 from the cytoplasm to the mitochondria plays a crucial role in propagating apoptotic signals to the cytoplasm [75,76]. On the contrary, the level of Bcl-2 was upregulated during autophagic cellular death, indicating that the autophagy process protects the cells from apoptotic death. It has been demonstrated, both in vivo and in vitro, that the autophagy process is mandatory for cell survival during starvation [77,78], which also protects epithelial cells from the apoptotic pathways [79] during metabolic stress [80]. Based on the aforementioned results, the activation of the autophagy process by drinking Zamzam water offers a unique chance for renal tubular epithelial cell survival in the face of cyclosporine-induced cellular apoptosis and nephrotoxicity. 

## 5. Conclusions 

The present study demonstrated that receiving ZW with the SC injection of CsA ameliorated its nephrotoxicity, as evidenced by the restoration of the renal function parameters and the subsequent improvement of the histological architecture. Additionally, ZW eliminated CsA-induced renal tubular apoptosis by decreasing the protein level of apoptotic markers and increasing the level of the antiapoptotic protein Bcl-2. Moreover, it enhanced the autophagy process by increasing LC3, Becline-1, and Atg5 while decreasing the P62 autophagic marker proteins, with a modulation of the AMPK/mTOR/ULK-1 pathway, thus restraining renal tubular cell apoptosis by boosting the autophagic process. 

## Figures and Tables

**Table 1:** Primer sequence of the examined genes.

	Forward Sequence (5′-3′)	Reverse Sequence (5′-3′)	Gene Accession Number
AMPK	AGCTCGCAGTGGCTTATCAT	GGGGCTGTCTGCTATGAGAG	NM_023991.1
mTOR	ACGAAGGAGACAGACCGAAG	CGACGAAGTCACTAGATTCA	AM_943028.1
GAPDH	CACCCTGTTGCTGTAGCCATATTC	GACATCAAGAAGGTGGTGAAGCAG	XM_017592435.1

**Table 2:** The levels of serum creatinine, blood urea nitrogen, and creatinine clearance in the experimental groups.

Groups	Serum Creatinine (mg/dL)	Blood Urea Nitrogen (mg/dL)	Creatinine Clearance Level (mL/min)
Control	0.68 ± 0.07	21.60 ± 4.60	0.248 ± 0.016
ZW	0.66 ± 0.08	22.80 ± 5.20	0.2520.018
CsA	1.99 ± 0.19 ***	90.40 ±7.15 ***	0.015 ± 0.003 ***
CsA + Zamzam water	0.94 ± 0.12 **^,*###*^	34.40 ± 4.42 ***^,*###*^	0.098 ± 0.021 ***^,###^

Values are displayed as mean ± SD. One-way ANOVA with Tukey’s analysis was used for comparison. *** *p* < 0.001, and, ** *p* < 0.001 vs. *control*; *^###^ p* <0.001 vs. CsA. Significant elevation vs. CsA group. CsA—cyclosporine A.

**Table 3:** Renal antioxidant enzymes and oxidative stress biomarkers in the experimental groups.

Classes	MDA (*nmol/g Tissue*)	SOD (*U/g/Tissue*)	CAT (*U/g/Tissue*)	GPx (*U/g/Tissue*)
control	11.33 ± 1.04	3.99 ± 0.19	2.20 ± 0.16	125 ±7.91
ZW	11.70 ± 0.93	4.15 ± 0.22	2.25 ± 0.15	128 ± 7.07
CsA	29.81 ± 3.41 ***	1.00 ± 0.13 ***	0.84 ± 0.09 ***	36.28 ± 5.67 ***
CsA+Zam	17.65 ± 1.83 ***^,###^	2.13 ± 0.38 ***^,###^	1.26 ± 0.22 ***^,###^	89.97 ± 7.14 ***^,###^

Data are presented as the mean ± SD. One-way ANOVA with Tukey’s analysis; *** *p* < 0.001 vs. *control*; *^###^ p* < 0.001 vs. CsA. MDA—malondialdehyde; SOD—superoxide dismutase; CAT—catalase; GPx—glutathione peroxidase.

**Table 4:** Physical and chemical analysis of Zamzam water.

Sample	ZW	Tap Water
Total hardness	670 mg/L	88 mg/L
Calcium hardness	422 mg/L	134 mg/L
Total alkalinity	302 mg/L	44 mg/L
Magnesium hardness	211 mg/L	77 mg/L
Calcium	189 mg/L	52 mg/L
Magnesium	520 mg/L	74 mg/L
Potassium	121 mg/L	12 mg/L
PH	7.8	7
Nitrite	0.01 mg/L	0.001 mg/L
Nitrates	173 mg/L	13 mg/L
Chlorine	343 mg/L	99 mg/L
Phosphates	0.27 mg/L	0.002 mg/L
Bicarbonate	364 mg/L	110 mg/L
Sulfites	370 mg/L	210 mg/L
Zinc	944 mg/L	186 mg/L
Selenium	320 mg/L	69 mg/L

## Data Availability

The data that support this research will be shared upon reasonable request to the corresponding authors.
